# Loving the love of boys: Motives for consuming yaoi media

**DOI:** 10.1371/journal.pone.0198895

**Published:** 2018-06-14

**Authors:** Ágnes Zsila, Dru Pagliassotti, Róbert Urbán, Gábor Orosz, Orsolya Király, Zsolt Demetrovics

**Affiliations:** 1 Institute of Psychology, ELTE Eötvös Loránd University, Budapest, Hungary; 2 Doctoral School of Psychology, ELTE Eötvös Loránd University, Budapest, Hungary; 3 California Lutheran University, Thousand Oaks, California, United States of America; 4 Institute of Cognitive Neuroscience and Psychology, Research Centre for Natural Sciences, Hungarian Academy of Sciences, Budapest, Hungary; University of Florence, ITALY

## Abstract

In recent years, yaoi has been increasingly popular among youth interested in Japanese media such as anime and manga. Yaoi is defined as commercial and fan-created media that thematically focus on the romantic love between two men, often in a sexually explicit way. Despite the widespread popularity of this Japanese subgenre, there is a lack of empirical studies on the motives for consuming yaoi media that analyze the full range of motives using reliable research methods. The present study aimed to explore and operationalize the motives for yaoi media consumption based on previous qualitative research (Pagliassotti, 2008). Using an online survey, 724 yaoi consumers (58% male; *M*_*age*_ = 30.1 years, *SD* = 10.4) completed the Yaoi Consumption Motives Questionnaire (YCMQ). According to confirmatory factor analyses, the bi-factor model of nine motives yielded closer fit to the data than the theoretically proposed, first-order ten-factor model and a second-order nine-factor model. The nine-factor YCMQ demonstrated strong psychometric properties in terms of factor structure, internal consistency, and measurement invariance. These results indicate that the 31-item YCMQ is an appropriate instrument to assess individuals’ motives for consuming yaoi media.

## Introduction

Japanese media (e.g., anime, manga) have been increasingly popular over the past two decades in the United States and several European countries [1]. Corresponding with this expansion, yaoi materials have attracted increasingly widespread interest among young women. Yaoi and boys’ love (BL) are umbrella terms for commercial and fan-created Japanese media (e.g., anime, comics, short stories, and artwork) that portray the romantic and erotic love between two men [1, 2]. Although yaoi is primarily targeted at young, heterosexual women, previous studies have found that bisexual and homosexual male and female enthusiasts also account for a considerable proportion of yaoi viewers/readers [3]. Yaoi viewers and readers often discuss original yaoi products and distribute fan-created yaoi materials in large online fan groups [4]. According to a recent survey conducted by the Yano Research Institute, the yaoi market (including commercial and fan-created products) generated 2.2 billion yen (24.5 million USD) in sales in 2010, and shows no signs of decay in recent years [1].

Homoerotic male/male manga created primarily by female artists for female consumers originated in the 1970s as a subgenre of shoujo, a manga genre specifically written for a younger female demographic. Terms for this new type of homoerotic manga that were used over the decades included *tanbi mono* (aesthetic fiction), *June mono* (fiction of the type published in *June* magazine), *bishōnen manga* (beautiful boy comics), *shōnen-ai* (*shōnen* = boys, and *ai* = love) [2] and *kunnaben riibe* (derived from the German *Knabenliebe*, or boy’s love) [1].

In the 1980s, Japanese fans began writing a form of amateur male/male homoerotic comics that romantically and sexually paired male characters who appeared in popular anime shows. This form of parody became known as “yaoi,” which is often said to be an acronym for “*yama nashi ochi nashi imi nashi*,” or “no climax, no ending, no meaning”—that is, manga focused on sexual scenarios, with little to no attention paid to plot or character arc [2]. Although similar to the earlier published works, yaoi manga appropriated copyright-protected characters and was often more humorous and explicit.

Yaoi became extremely popular in Japan’s amateur manga markets and spread to other countries over the course of the 1990s, largely due to fan communities sharing unlicensed copies of the comics and translations via the internet. For example, researchers have documented that BL manga was mentioned in China in the early 1990s [1] and in South Korea by the late 1990s [5]. In the United States, the term “yaoi” was used to encompass all forms of this manga; an annual fan convention calling itself “Yaoi-Con” was founded in 2001 and continues to the present day, helping to cement the use of “yaoi” within the U.S.

Meanwhile, in Japan, the term “*bōizu rabu*” (a term derived from boy’s love, the English translation of *shōnen-ai*) was adopted by some Japanese publications [2], as was use of “boy’s love” or “BL” itself [1]. Adoption of the term “boy’s love” to replace “yaoi” was slower in the United States, where “boy’s love” suggests pedophilia; publishers generally did not adopt the term in their imprint names.

Although researchers have attempted to differentiate between different types of male/male manga subgenres, fans use the terms interchangeably. Suzuki [6] interviewed several professional Japanese BL writers and identified five subgenres: *shōnen’ai*, *tanbi*, *JUNE*, *yaoi*, and *boys love*. However, she simultaneously noted that the writers tended to use “yaoi” as a catch-all term because “there is no appropriate and convenient Japanese shorthand term to embrace all subgenres of male-male love fiction by and for women” (p. 109). Nagaike and Aoyama [7] avoid attempts at differentiation, pointing out that “many BL researchers use ‘BL’ or, less frequently, ‘*yaoi’* as umbrella terms” (p. 120) because the various genre categories have never been clearly defined and, even when subgenres are differentiated, “they remain thematically intertwined” (p. 120). The present research, originating from a survey created in the United States at a time when “yaoi” was the accepted term among fans, and given again in Hungary, where “yaoi” continues to be the most popular term used by fans, employs “yaoi” as an umbrella term to encompass all forms of boys’ love media while acknowledging the historical differences in nomenclature.

The increasing popularity of yaoi media has received attention in the motivation literature of several interdisciplinary fields (e.g., gender studies, media psychology, cultural anthropology) [8]. Motivation can be described as a process that manifests in the maintenance of desired or undesired physical and psychological activities and in the persistence of behavioral responses [9]. Although several studies have investigated the reasons underlying the global popularity of yaoi media [4, 10, 11], empirical research into yaoi viewing/reading motives is scarce.

Pagliassotti [12, 13] conducted one of the earliest surveys on yaoi viewing/ reading motives of Anglophone yaoi consumers outside of Japan. According to the results of her qualitative research, ten distinct motive dimensions could be identified. The first, *“Pure” love without gender* dimension refers to a motive to view/read romantic contents in which gender differences are not present. The *Pro-gay attitude/forbidden & transgressive love* dimension represents a motive to view/read materials that portray a positive picture of homosexual men. *Identification/self-analysis* refers to yaoi viewers’ and readers’ motive to better understand their own feelings and dilemmas. The *Melodramatic/emotional elements* dimension represents a motive to experience intense emotions. *Dislike for standard romances/shoujo* reflects a motive to avoid heterosexual romance stories (e.g., shoujo) due to their often one-dimensional female characters and ‘boring’ narratives. *A female-oriented romantic/erotic genre* dimension refers to a motive to consume yaoi because it caters to women’s narrative preferences. *Pure escapism/lack of reality* reflects a motive to escape daily life. *Art and aesthetics* represents a motive to view/read yaoi for its distinctive graphic style. The *Pure entertainment* dimension refers to a motive of seeking relaxation and entertainment. Finally, *Arousing/sexually titillating* reflects a motive of seeking sexually arousing content.

These diverse motivations for consuming yaoi may be complicated by cultural and legal differences between various countries that affect the context in which yaoi is consumed. For example, female readers may consume yaoi as part of their rejection of or resistance to conservative patriarchal or parental norms about sex and gender [5, 14, 15]— as might some male readers [16]. In other cultures, fans may read yaoi as a way of affirming and/or participating in gay rights communities [13, 17], rejecting gender binarism [18], or enjoying romance narratives that avoid problematic female stereotypes [19].

In addition, a nation’s laws may determine which kinds of yaoi are legally available to consumers in any particular country, further affecting consumers’ motivations to read the works. For example, licensed Japanese yaoi in the United States are chosen and edited to avoid legal or social condemnation [20] while yaoi consumers in Australia risk arrest if they choose to read manga or watch anime about characters who appear to be under the age of sexual consent [21]. Yaoi creators and consumers face social and/or legal pressures in Indonesia [22], Taiwan [23], and China [14, 15]. Consumers’ motives for consuming a medium that is highly regulated or even illegal are likely to differ in some respects from the motives of those who can consume the medium without taking any legal risk.

Recently, Chou [24] conducted a qualitative survey on Taiwanese yaoi readers and found that female readers are motivated to challenge social taboos, experience freedom from gender constraints, and enjoy incorporating traditional gender norms into homosexual love stories while reading or creating yaoi. Similarly, Lilja and Wasshede [18] found that Swedish yaoi readers expressed an interest in yaoi materials for their portrayal of gender equality and their sexually arousing aspects. Zhang [14] investigated young female Chinese yaoi enthusiasts’ motives and found that challenging traditional gender constructs, escapism, voyeurism, and aesthetics are the most attractive features of yaoi media for women. Nagaike [16] analyzed the motives of male heterosexual yaoi enthusiasts and concluded that male viewers/readers wish to identify with feminized male characters in narratives that deconstruct the social paradigms of masculinity, thus gaining a deeper understanding of their own dilemmas relating to gender conventions.

However, despite offering insight into the yaoi consumption motives of culturally diverse viewers/readers, most of these studies did not test their assertions quantitatively and did not use methods that could provide future researchers with an accurate and reliable assessment instrument. Furthermore, the majority of previous studies have focused specifically on yaoi *manga* reading motives instead of exploring yaoi media consumption motives comprehensively.

Therefore, the present study aimed to explore the motivational background of yaoi media consumption. Drawing on the comprehensive motivational framework suggested by Pagliassotti [13], the primary goal of the present investigation was to identify the range of motivational factors prompting yaoi media consumption. The secondary aim of this study was to operationalize these dimensions, develop a scale to assess the identified factors, and analyze the psychometric properties of the constructed assessment instrument.

## Materials and methods

### Participants and procedure

Data collection was carried out using an online survey focusing on sexual behaviors and pornography consumption habits. A call for voluntary participation was advertised on one of the most visited Hungarian news websites (444.hu). As an incentive, two tablets were raffled off among all participants who completed the online survey. Before completing the questionnaire, participants were informed about the study’s aims and were asked to provide informed consent by ticking a box if they were over 18 years old and agreed to participate in the survey.

A total of 24,372 participants (68.52% male, *M*_*age*_ = 33.45 years, *SD* = 11.36) started to complete the questionnaire. In accordance with the object of the present study, further data analysis was conducted on a sample of participants who read or viewed yaoi within the past year (4.42% of the total sample). Participants who did not complete the yaoi motives questionnaire (19.94% of the yaoi viewer subsample), provided inconsistent answers (1.21%), or gave the same response consistently to each questionnaire item (11.69%) were excluded from the analysis. Therefore, the final sample comprised 724 participants aged 18–73 years (58.01% male; *M*_*age*_ = 30.09 years, *SD* = 10.42). Less than half of the participants reported being heterosexual (43.23%); 20.30% reported being heterosexual with homosexuality to some extent; 13.54% reported being bisexual, 4.01% reported being homosexual with heterosexuality to some extent, 14.09% reported being homosexual, 0.28% reported being asexual, 2.76% reported being unsure about their sexual orientation, and 1.80% indicated ‘other’.

### Measures

Data regarding major demographic characteristics (gender, age, and sexual orientation) were collected. In further analysis, sexual orientation was dichotomized in the following way: 0 = self-reported heterosexuals and heterosexuals with homosexuality to some extent (i.e., ‘heterosexuals’) (*n* = 460; 53.91% male); 1 = self-reported bisexuals, homosexuals with heterosexuality to some extent, homosexuals, asexuals, and those participants who reported being unsure about their sexual orientation, or used the ‘other’ category to describe their sexual preferences (i.e., ‘non-heterosexuals’) (*n* = 264; 65.15% male).

Participants read a definition of yaoi based on a description by Welker [1]:*“Yaoi (also known as Boys’ Love [BL]) comprise Japanese media (e*.*g*., *anime*, *manga) that are centered on romantic or sexual relationship between men*.*”* Participants who responded positively to the question of whether they had viewed/read yaoi within the past year were also asked to indicate the frequency of their yaoi viewing/reading and related creative activities (i.e., writing stories, drawing artwork, making videos); their age at first exposure to this genre, and their motives for these activities. The yaoi viewing/reading and creating variables were dichotomized in the following way: 1 = rare yaoi viewing/reading or creating (i.e., viewed/read or created yaoi 1–11 times in the past year), and 2 = frequent yaoi viewing/reading or creating (i.e., viewed/read or created yaoi at least monthly in the past year).

### Item construction

The aim of the present study was to explore the underlying motives for yaoi media consumption. To achieve this goal, a multidimensional instrument was developed that assesses yaoi viewing/reading motives. Based on the ten motivational dimensions identified by Pagliassotti [13] in her qualitative research, three or four items were constructed per factor, resulting in a 31-item pool. The statements were created to reflect the content of the original responses in the report of Pagliassotti [13] as closely as possible. The most typical responses were considered as the basis of selection.

Participants were asked to indicate to what extent each of the 31 statements was characteristic of them (“I view/read yaoi…”) on a five-point Likert scale (ranging from 1 = very uncharacteristic of me to 5 = very characteristic of me). The ten motivational dimensions, based on the findings of Pagliassotti [13], were as follows: “Pure” love without gender (three items; e.g., “because the partners are equal in it”), Pro-gay attitude/forbidden & transgressive love (three items; e.g., “because gay men have equal rights in it”), Identification/self-analysis (three items; e.g., “because it gives me an opportunity to better understand my feelings”), Melodramatic/emotional elements (three items; e.g., “because it evokes deep emotions in me”), Dislike for standard romances/shoujo (three items; e.g., “because heterosexual romance is boring”), A female-oriented romantic/erotic genre (four items; e.g., “because it expresses erotica in a way that is more enjoyable for women”), Pure escapism/lack of reality (three items; e.g., “because I can temporarily escape from reality”), Art and aesthetics (three items; e.g., “because it is aesthetic”), Pure entertainment (three items; e.g., “because it is entertaining”), Arousing/sexually titillating (three items; e.g., “because sex between men is arousing for me”).

### Statistical analysis

Descriptive statistics were explored using IBM SPSS version 22.0 (IBM SPSS Inc., Chicago, Illinois). Effect size indices (Cohen’s *d*) were calculated with 0.1 being interpreted as a small effect, 0.3 as a medium effect, and 0.5 as a large effect [25].

To test the hypothesized ten-factor structure of the constructed scale, confirmatory factor analysis (CFA) was performed with Mplus 8.0 [26] using a weighted least squares mean- and variance-adjusted estimator (WLSMV) within a structural equation modeling (SEM) framework. Items were treated as ordinal indicators due to severe floor effect in some items and serious deviation from normal distribution in the responses. For the CFA, the following fit indices were used to evaluate the goodness of fit of the tested model [27, 28]: the Comparative Fit Index (CFI; ≥ 0.95 for good, ≥ 0.90 for acceptable), the Tucker-Lewis index (TLI; ≥ 0.95 for good, ≥ 0.90 for acceptable), and the Root-Mean-Square Error of Approximation (RMSEA; ≤ 0.06 for good, ≤ 0.08 for acceptable), with its 90% confidence interval. Internal consistency was assessed using Cronbach’s alpha, with values above 0.70 being interpreted as acceptable and values above 0.80 being interpreted as good [29].

Besides the first-order model of primary factors, two higher-order constructs were tested: a second-order model and a bi-factor model. Acknowledging the high correlations among primary factors, the second-order model served to offer explanation for the inter-factor correlations.

Estimating a bi-factor measurement structure alongside a second-order model has been proposed as an effective approach to exploring construct-relevant multidimensionality [30]. The bi-factor measurement model allows for the indicators of motives to load on an overall primary factor (e.g., a general preference for the yaoi genre) and to have a secondary loading on specific motive dimensions. The general specification of a bi-factor model requires that the specific factors correlate neither with each other nor with the general factor. Therefore, correlations between specific factors and the general factor were fixed to zero [31]. In order to compare the two higher-order models, a DIFFTEST procedure (implemented in Mplus) was performed, which allows for the comparison between nested models with ordinary indicators [26].

To ensure that group comparisons are meaningful when investigating differences between men and women, heterosexuals and non-heterosexuals in yaoi consumption motives, measurement invariance of the retrained bi-factor model was tested across gender and sexual orientation with the convenience feature of Mplus using delta parameterization [26]. Thus, several multigroup CFAs were performed [32–34]. In the first step, two separate models were estimated freely for male and female and heterosexual and non-heterosexual participants. Subsequently, configural invariance was tested in which factor loadings and thresholds were free across groups, scale factors were fixed at one in all groups, and factor means were fixed at zero in all groups. In the next step, metric invariance (i.e., invariance of factor loadings) was investigated in most studies. However, when analyzing categorical outcomes in an invariance framework in Mplus, thresholds and factor loadings must be freed or constrained, which cannot be applied to a test of metric invariance. Therefore, after the configural model, scalar invariance was tested in which factor loadings and thresholds were constrained to be equal across groups, scale factors were fixed atone in one group and held free in the other groups, and factor means were fixed at zero in one group and were freed in the other groups. When comparing the increasingly constrained models, relative change in the fit indices (i.e., ΔCFI and ΔRMSEA) was also examined. The following fit indices were used when comparing the increasingly constrained models [35–37]: ΔCFI ≤ 0.010; ΔTLI ≤ 0.010; and ΔRMSEA ≤ 0.015.

In the final step, a multiple indicators multiple causes (MIMIC) confirmatory factor analysis was conducted on the retained bi-factor model to investigate the associations between yaoi motives, gender, age, and sexual orientation. MIMIC models allow estimation of the effect of indicators on latent variables while controlling for the direct effect of grouping variables on latent constructs. In the present study, the MIMIC model incorporates the yaoi motive dimensions as latent factors and the respective items as indicators of latent variables. This part of the model is equivalent to a confirmatory factor analysis. However, the model is complemented with exogenous variables (i.e., gender, age, and sexual orientation) in the present study, which allows for the investigation of motivational differences across gender, age, and sexual orientation.

### Ethics

This study was approved by the Institutional Review Board of ELTE Eötvös Loránd University and was carried out in accordance with the Declaration of Helsinki. All participants were informed about the aim of the study and provided written consent before completing the questionnaire.

## Results

### Descriptive statistics

The majority of participants (71.13%) reported viewing/reading yaoi occasionally during the past year, whereas 28.87% viewed/read yaoi monthly or more frequently (see Table 1). Using χ^2^ tests, it was found that frequent yaoi viewers/readers were more likely to be female than male (32.9% vs. 26.0%, χ^2^(1) = 4.14; *p* = 0.05; *Phi* = 0.08). Likewise, the proportion of frequent yaoi viewers/readers was higher among non-heterosexuals than among heterosexuals (36.4% vs. 24.6%, χ^2^(1) = 11.37; *p* = 0.001; *Phi* = 0.13). The frequency of yaoi viewing/reading decreased with age (*r* = -0.14; *p* < 0.001).

**Table 1:** Descriptive statistics of yaoi viewing/reading and creating activities among participants (*N* = 724).

	Frequency of viewing/reading yaoi	Frequency of creating yaoi
Never	—	635 (87.71%)
1–6 times in the past year	452 (62.43%)	49 (6.77%)
7–11 times in the past year	63 (8.70%)	7 (0.97%)
Once per month	54 (7.46%)	5 (0.69%)
2–3 times per month	64 (8.84%)	4 (0.55%)
Once per week	21 (2.90%)	1 (0.14%)
2–3 times per week	26 (3.59%)	7 (0.97%)
4–5 times per week	14 (1.93%)	6 (0.83%)
6–7 times per week	30 (4.14%)	10 (1.38%)

*Note*. The number of participants is reported for the frequencies of viewing/reading and creating yaoi with the respective percentages in parenthesis.

In the present sample, the vast majority of participants (87.71%) did not create yaoi materials in the past year, and a considerable proportion of participants who engaged in yaoi-related creative activities created yaoi materials relatively rarely (see Table 1 for details).

The average age of participants at their first exposure to yaoi was 13.77 years (*SD* = 7.82). Female participants were introduced to yaoi at younger ages (*M* = 12.01, *SD* = 5.98) than male participants (*M* = 15.04, *SD* = 8.70) (*t*(699) = 5.47; *p* < 0.001; *d* = 0.39). The effect size was medium. However, there was no significant difference in the mean age at first exposure to yaoi between heterosexuals (*M* = 14.04, *SD* = 8.21) and non-heterosexuals (*M* = 13.31, *SD* = 7.08) (*t*(700) = 1.20; *p* = 0.23).

### Measurement models of the Yaoi Consumption Motives Questionnaire (YCMQ)

#### Confirmatory factor analysis (CFA)

Confirmatory factor analysis was performed on the original 31-item pool applying the theoretically grounded ten-factor model (*N* = 724). The fit indices showed an adequate fit to the data (*χ*^*2*^ = 1823.38; *df* = 389; *p* < 0.001; CFI = 0.957; TLI = 0.948; RMSEA = 0.071 [0.068–0.075]). Factor loadings and descriptive statistics are presented in Table 2.

**Table 2:** Items, descriptive statistics, and reliability indices of the Yaoi Consumption Motives Questionnaire (YCMQ) (*N* = 724).

I view/read yaoi…	α	Descriptive statistics		Factor loadings										
		Mean	SD	CFA_1_	CFA_2_	Bi-factor model		Reliability indices of specific factors bi-factor model						
				Primary factor (SE)	Primary factor (SE)	Specific factor (SE)	General factor* (SE)	ECV	Ω				Ωh	H
“Pure” love without gender & pro-gay attitude	0.87	2.35	1.07					0.04	0.91				0.19	0.54
1. … because the partners are equal in it				0.67 (0.03)	0.67 (0.03)	0.33 (0.03)	0.59 (0.03)							
2. … because there are no gender differences in it				0.81 (0.02)	0.81 (0.02)	0.32 (0.04)	0.73 (0.02)							
3. … because feelings have priority over gender in it				0.88 (0.02)	0.89 (0.01)	0.24 (0.03)	0.81 (0.02)							
4. … because this genre breaks social taboos				0.76 (0.02)	0.73 (0.02)	0.28 (0.04)	0.65 (0.02)							
5. … because gay men have equal rights in it				0.86 (0.02)	0.82 (0.02)	0.62 (0.04)	0.70 (0.02)							
6. … because this genre portrays a positive picture of gay men				0.86 (0.02)	0.83 (0.02)	0.38 (0.03)	0.74 (0.02)							
Identification/self-analysis	0.87	1.68	0.97					0.03	0.94				0.27	0.49
7. … because it helps me better understand my life events				0.88 (0.02)	0.88 (0.02)	0.50 (0.03)	0.74 (0.02)							
8. … because it gives me an opportunity to better understand my feelings				0.96 (0.01)	0.96 (0.01)	0.45 (0.03)	0.82 (0.02)							
9. … because it provides me with a guide to better understand my sexual dilemmas				0.88 (0.02)	0.88 (0.02)	0.51 (0.03)	0.74 (0.02)							
Melodramatic/emotional elements	0.86	2.20	1.23					0.01	0.92				0.09	0.21
10. … because it portrays intense feelings				0.86 (0.01)	0.86 (0.01)	0.20 (0.04)	0.82 (0.02)							
11. … because it has a stronger emotional impact on me than other stories				0.89 (0.01)	0.89 (0.01)	0.30 (0.05)	0.84 (0.02)							
12. … because it evokes deep emotions in me				0.92 (0.01)	0.92 (0.01)	0.33 (0.05)	0.88 (0.01)							
Dislike for standard romances/shoujo	0.75	1.95	1.02					0.03		0.86			0.22	0.42
13. … because classic female protagonists are one-dimensional				0.83 (0.02)	0.83 (0.02)	0.23 (0.04)	0.73 (0.02)							
14…. because heterosexual romance is boring				0.76 (0.02)	0.76 (0.02)	0.54 (0.07)	0.64 (0.03)							
15…. because traditional romance is out of fashion				0.84 (0.02)	0.84 (0.02)	0.46 (0.06)	0.72 (0.02)							
A female-oriented romantic/erotic genre	0.90	1.96	1.06					0.06		0.94			0.38	0.66
16. … because it is specifically made for women				0.78 (0.02)	0.78 (0.02)	0.58 (0.03)	0.58 (0.03)							
17. because it expresses erotica in a way that is more enjoyable for women				0.92 (0.01)	0.92 (0.01)	0.57 (0.03)	0.71 (0.02)							
18. … because its romanticism is closer to women’s preferences				0.91 (0.02)	0.91 (0.02)	0.49 (0.03)	0.72 (0.02)							
19. … because it portrays sexuality in an appropriate way for women				0.92 (0.01)	0.92 (0.01)	0.61 (0.03)	0.71 (0.02)							
Pure escapism/lack of reality	0.86	1.90	1.10					0.03			0.93		0.21	0.42
20. … because I can avoid reality				0.85 (0.02)	0.85 (0.02)	0.53 (0.04)	0.74 (0.02)							
21. … because I can temporarily escape from reality				0.90 (0.01)	0.90 (0.01)	0.37 (0.03)	0.80 (0.02)							
22. … because it helps me to forget about daily hassles				0.93 (0.01)	0.93 (0.01)	0.38 (0.03)	0.82 (0.02)							
Art and aesthetics	0.82	2.77	1.23					0.03			0.88		0.31	0.54
23…. because it is aesthetic				0.79 (0.02)	0.79 (0.02)	0.39 (0.04)	0.65 (0.02)							
24…. because erotica is artistically portrayed in it				0.93 (0.01)	0.93 (0.01)	0.43 (0.03)	0.75 (0.03)							
25…. because I like its graphics				0.78 (0.02)	0.78 (0.02)	0.66 (0.04)	0.62 (0.02)							
Pure entertainment	0.80	2.60	1.14					0.02				0.87	0.20	0.42
26…. because it is entertaining				0.76 (0.02)	0.76 (0.02)	0.28 (0.05)	0.67 (0.02)							
27…. because it fills my free time				0.84 (0.02)	0.84 (0.02)	0.31 (0.05)	0.74 (0.02)							
28…. because it is relaxing to me				0.87 (0.01)	0.87 (0.01)	0.59 (0.07)	0.77 (0.02)							
Arousing/sexually titillating	0.80	2.86	1.25					0.06				0.87	0.58	0.73
29. … because it sexually arouses me				0.66 (0.03)	0.66 (0.03)	0.76 (0.03)	0.34 (0.04)							
30. … because sex between men is arousing for me				0.91 (0.02)	0.91 (0.02)	0.69 (0.03)	0.54 (0.03)							
31. … because I can act out my secret sexual desires through it				0.89 (0.03)	0.89 (0.03)	0.56 (0.03)	0.55 (0.03)							

*Notes*.α = Cronbach’s alpha value; SD = standard deviation; CFA = confirmatory factor analysis.

The instructions were as follows: People view/read yaoi for different reasons. Some reasons are listed below. Please indicate to what extent each of the following statements is characteristic of you by clicking on the appropriate response: 1 –very uncharacteristic of me, 2 –slightly characteristic of me, 3 –moderately characteristic of me, 4 –characteristic of me, 5 –very characteristic of me.

The column of CFA_1_ represents factor loadings yielded in the preliminary CFA conducted on the original ten motive factors, whereas the column of CFA_2_ represents factor loadings yielded in the investigation of the final, nine-factor structure of the YMCQ.

In the bifactor model correlations among specific factors as well as between general and specific factors were fixed to zero. ECV indicates the proportion of common variance explained by the target construct. Ω (omega) refers to the proportion of explained variance in the scale score attributed to the global and the specific factors. Ωh (omega hierarchical) refers to the proportion of explained variance of the scale score attributed to the specific factor. H represents the proportion of variability in the construct explained by its own indicator variables. *Reliability indices of the general factor are ECV = 0.70, Ω = 0.98, Ωh = 0.94, H = 0.97, and PUC = 0.91.

Correlations between latent factors were generally high (Mean_r_ = 0.69; max_r_ = 0.93 min_r_ = 0.34), indicating the possibility of a higher-order factor structure. The strongest association was observed between the “Pure” love without gender and the Pro-gay attitude/forbidden & transgressive love (*r* = 0.93; *p* < 0.001) factors. As a result of this extremely high correlation, “Pure” love without gender and Pro-gay attitude/forbidden & transgressive love motive dimensions were merged into one factor called “Pure” love without gender and pro-gay attitude. Therefore, nine motive factors were analyzed in the subsequent test of second-order and bi-factor models. Similarly strong correlations were found between Melodramatic/emotional elements and “Pure” love without gender (*r* = 0.86; *p* < 0.001), and Pure entertainment and Art and aesthetics (*r* = 0.87; *p* < 0.001).

#### The second-order model

In order to seek explanation for the high correlations among all motive factors, two higher-order factor models were also estimated: a second-order model with one second-order factor and a bi-factor model that specifies both a single common factor and nine specific factors. The second order/general factor served to provide explanation for the high correlations between primary factors. This secondary factor (in which all motive factors contribute to a general motive factor) may indicate the consumer’s general preference for the yaoi genre.

In the second-order factor model, all primary factors loaded on one second-order factor. The estimation yielded an acceptable degree of fit (*χ*^*2*^ = 2162.7; *df* = 425; *p* < 0.001; CFI = 0.948; TLI = 0.943; RMSEA = 0.075 [0.072–0.078]). The factor loadings of primary factors ranged between 0.59 and 0.95.

#### The bi-factor model

In addition to the second-order model, the impact of general attitude factor was estimated on each item. More specifically, the bi-factor measurement model allowed the indicators of motives to load on an overall primary factor (i.e., the general factor of preference for the yaoi genre) and to have a secondary loading on the specific motive dimensions. As the general specification of a bi-factor model requires that the specific factors correlate neither with each other nor with the general factor [30], test of the bi-factor model was performed without allowing the covariance among specific factors. The degree of model fit was adequate (*χ*^*2*^ = 1955.1; *df* = 403; *p* < 0.001; CFI = 0.953; TLI = 0.946; RMSEA = 0.073 [0.070–0.076]). In comparison with the second-order factor model, the bi-factor model yielded a closer fit to the data (Δχ^2^ = 262.9, Δdf = 22, p < 0.001).

**Fig 1:**
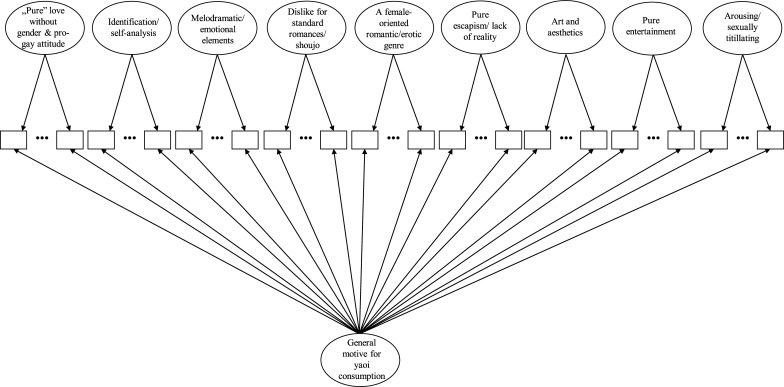
Schematic representation of the bi-factor model of the Yaoi Consumption Motives Questionnaire (YCMQ). Psychometric properties of the bi-factor model are presented in Table 2.

Standardized factor loadings of this bi-factor model are presented in Table 2. All items loaded on the general factor with salient loadings (> 0.34, mean of loadings = 0.71 SD = 0.11). On the specific factors, loadings were much more varied (between 0.20 and 0.76). The bi-factor model is presented in Fig 1.

Relevant reliability indices including explained common variance, omega, omega hierarchical, and H index of the general and specific factors were also estimated. Omega values (ranging from 0.86 to 0.94) indicate that the variance is largely explained by the general and the specific factors. However, the omega hierarchical values were much smaller in specific factors (ranging from 0.09 to 0.58), indicating that the contribution of specific factors to the total variance is much smaller than the general factor’s. Therefore, much of the variance of each specific score is explained largely by the general factor, and only moderately or slightly by the specific factors. The Melodramatic/emotional elements factor yielded the weakest contribution, whereas Arousing/sexually titillating, A female-oriented romantic/erotic genre, Art and aesthetics, and Identification/self-analysis factors provided the largest contribution.

### Testing measurement invariance across gender and sexual orientation

Results of the measurement invariance analysis based on the bi-factor model are presented in Table 3. Baseline models for male and female participants indicated a good fit to the data. When parameters were constrained, changes in fit indices were observed. With regard to gender invariance, fit indices for the configural model demonstrated an acceptable fit to the data (*χ*^*2*^ = 2388.0; *df* = 806; *p* < 0.001; CFI = 0.948, TLI = 0.940, RMSEA = 0.074 [0.070–0.077]). Subsequently, a scalar model was estimated, resulting in a negligible change in the fit indices (ΔCFI = -0.004, ΔTLI = 0.005, ΔRMSEA = -0.004). Therefore, measurement invariance was demonstrated based on gender, indicating that group comparisons across gender are meaningful when discussing yaoi viewing/reading motives.

**Table 3:** Tests of invariance in gender and sexual orientation on the bi-factor model of yaoi consumption motives.

Model	χ^2^	df	CFI	TLI	RMSEA (90% CI)	Model comparison	Δχ^2^ (df)	∆CFI	∆TLI	∆RMSEA
Gender										
Baseline male (*n* = 420)	1449.6***	403	0.939	0.930	0.079 (0.074–0.083)					
Baseline female (*n* = 304)	959.2***	403	0.957	0.950	0.067 (0.062–0.073)					
Configural (unconstrained model)	2388.0***	806	0.948	0.940	0.074 (0.070–0.077)					
Scalar (constrained model)	2621.1***	941	0.944	0.945	0.070 (0.067–0.073)	unconstrained vs. constrained model	361.6 (135)***	-0.004	0.005	-0.004
Sexual orientation										
Baseline heterosexual (*n* = 460)	1295.6***	403	0.957	0.951	0.069 (0.065–0.074)					
Baseline non-heterosexual (*n* = 264)	899.0***	403	0.958	0.952	0.068 (0.062–0.074)					
Configural (unconstrained model)	2171.7***	806	0.958	0.952	0.068 (0.065–0.072)					
Scalar (constrained model)	2453.1***	941	0.954	0.955	0.067 (0.063–0.070)	unconstrained vs. constrained model	391.2 (135)***	-0.004	0.003	-0.001

Notes. χ2 = chi-square; df = degrees of freedom; CFI = comparative fit index; TLI = Tucker-Lewis index; RMSEA = root mean square error of approximation; ΔCFI = change in CFI value compared to the preceding model; ΔTLI = change in the TLI value compared to the preceding model; ΔRMSEA = change in the RMSEA value compared to the preceding model.

***p < 0.001.

Measurement invariance was also tested with regard to sexual orientation. Again, fit indices for the configural model had an acceptable fit to the data (*χ*^*2*^ = 2171.7; *df* = 806; *p* < 0.001; CFI = 0.958, TLI = 0.952, RMSEA = 0.068 [0.065–0.072]). After the subsequent test of scalar invariance, again a negligible chance could be observed in the fit indices (ΔCFI = -0.004, ΔTLI = 0.003, ΔRMSEA = -0.001). Therefore, measurement invariance was also demonstrated with regard to sexual orientation, indicating that group comparisons across heterosexual and non-heterosexual yaoi consumers are meaningful in terms of yaoi viewing/reading motives.

### Multiple indicator multiple cause model (MIMIC) on demographics and yaoi consumption motives

The effect of gender, age, and sexual orientation on yaoi viewing/reading motives was investigated by adding three exogenous observed variables (i.e., gender, age, and sexual orientation) to the bi-factor CFA model. This complemented model had a good fit to the data (*χ*^*2*^ = 1993.10; *df* = 466; *p* < 0.001; CFI = 0.947, TLI = 0.937, RMSEA = 0.067 [0.064–0.070]). The psychometric properties of this model are presented in Table 4.

**Table 4:** Predictors of motivational factors: A multiple indicator multiple cause model (MIMIC).

Outcome variables	Explanatory variables beta (SE)			R^2^
Motivational factors	Gender	Age	Sexual orientation	
General factor	**0.38** (0.04)	0.02 (0.05)	0.04 (0.05)	14%
Pure love without gender & pro-gay attitudes	0.07 (0.06)	-0.03 (0.06)	**0.52** (0.05)	27%
Identification/self-analysis	**-0.41** (0.07)	-0.04 (0.06)	**0.41** (0.06)	37%
Melodramatic/ emotional elements	0.04 (0.09)	**-0.26** (0.08)	**0.57** (0.07)	41%
Dislike for standard romances/shoujo	-0.12 (0.07)	-0.12 (0.06)	**0.40** (0.06)	20%
A female-oriented romantic/erotic genre	**0.31** (0.06)	0.05 (0.05)	-0.08 (0.06)	10%
Pure escapism/ lack of reality	**-0.18** (0.08)	-0.09 (0.07)	**0.25** (0.08)	11%
Art and aesthetics	0.04 (0.06)	0.02 (0.06)	**0.30** (0.06)	9%
Pure entertainment	0.06 (0.08)	-0.02 (0.06)	**0.30** (0.07)	9%
Arousing/ sexually titillating	**-0.12** (0.05)	0.01 (0.05)	**0.48** (0.04)	26%

*Note*. Boldfaced coefficients are significant at least at *p* < .05. Gender is coded as 0 = males and 1 = females. Sexual orientation was coded as 0 = heterosexuals, and 1 = homosexuals. Age was a continuous variable.

With regard to gender differences, female participants scored higher than male participants on the general motive along with one specific yaoi viewing/reading motive dimension: A female-oriented romantic/erotic genre (*β* = 0.38; *p* < 0.001). By contrast, male participants yielded higher scores than female participants on three specific factors: Identification/self-analysis (*β* = -0.41; *p* < 0.001), Pure escapism/lack of reality (*β* = -0.18; *p* = 0.03) and Arousing/sexually titillating (*β* = -0.12; *p* = 0.01). Only one motivational dimension showed a weak, negative association with age: Melodramatic/ emotional elements (*β* = -0.26; *p* = 0.001), indicating that this motive decreases with increasing age. Non-heterosexuals scored higher than heterosexuals on all yaoi viewing/reading motives except for A female-oriented romantic/erotic genre (*β* = -0.08; *p* = 0.19) and the general motive dimension (*β* = 0.07; *p* = 0.11).

## Discussion

The present study investigated the motives behind yaoi media consumption. Based on the qualitative survey of Pagliassotti [13], a comprehensive, ten-factor model was tested to identify and operationalize a range of yaoi viewing/reading motives. Although confirmatory factor analysis supported the proposed ten-factor model, two latent motives were merged into one single factor due to their extremely high correlation (above 0.9). Therefore, the nine dimensions were the following: “Pure” love without gender & pro-gay attitude, Identification/self-analysis, Melodramatic/emotional elements, Dislike for standard romances/shoujo, A female-oriented romantic/erotic genre, Pure escapism/lack of reality, Art and aesthetics, Pure entertainment, and Arousing/sexually titillating.

Due to several highly correlated factors, two higher-order models were also tested: a second-order model with one second-order factor and a bi-factor model that specified both a general factor and nine specific factors. The second-order factor in the background of the original factors may indicate a general preference for the yaoi genre. More specifically, fans of this genre may tend to indicate high levels of motivation in general as a result of their dedication to the genre. Both higher-order models had acceptable fit with the bi-factor model yielding the closest fit to the data. Therefore, the bi-factor structure was retained and used in subsequent analyses.

The Yaoi Consumption Motives Questionnaire (YCMQ) with its nine motivational dimensions allows for the identification, description, and operationalization of a considerably comprehensive motivational profile of the yaoi genre consumer. Furthermore, the YCMQ demonstrated strong psychometric properties in terms of factor structure, reliability, and measurement invariance.

Furthermore, the application of a bi-factor model allowed for the estimation of the role of a general factor alongside with relevant specific factors that represent each motive dimension. The variance in scores of each motive factor is explained largely by a general motive factor, with the exception of some factors in which specific factors also had relevant contribution. According to this, the most important specific factors are Arousing/sexually titillating, Art and aesthetics, A female-oriented romantic/erotic genre, and Identification/self-analysis. In addition, the factors of “Pure” love without gender & pro-gay attitude, Dislike for standard romances/shoujo, Pure escapism/lack of reality, and Pure entertainment still explained around 20% of the variance out of the specific factor scores besides the general motivational factor. However, Melodramatic/emotional elements factor score largely reflects the general motive factor. The general score of all items could be a reliable reflection of a general positive attitude towards this genre. Future research should pay attention to the implication of these findings when applying this scale for the assessment of a general positive attitude towards yaoi as well as each specific motive dimension related to this genre.

The motive dimensions of Arousing/sexually titillating and Art and aesthetics represented a considerable proportion of variance in the present study, suggesting that the most attractive features of yaoi media for yaoi viewers/readers are their sexual content and their aesthetic portrayal of characters and sexuality. These motives were noted in early studies by Mizoguchi [2] and Thorn [4], as well as in more recent studies [14, 18], further confirming the special importance these aspects of yaoi media hold for their viewers/ readers. Furthermore, male viewers/readers yielded higher scores than females in the Arousing/sexually titillating motive dimension. This result is in line with previous studies that found men having higher motivation to seek sexual content on the Internet than women [38, 39].

In addition, A female-oriented romantic/erotic genre and Identification/self-analysis were found to be important motive dimensions in the assessment. A female-oriented romantic/erotic genre was especially important to the female survey participants supporting previous qualitative research indicating that yaoi consumers find that it offers a means of avoiding or negotiating the gender stereotypes [18, 19], or patriarchal messages [5, 14, 24] that may occur in other forms of romantic narrative. In a similar vein, female yaoi viewers/readers scored higher than male consumers on the general motive for yaoi consumption, indicating that female consumers may find yaoi media more inspiring, entertaining and close to their preferences than male consumers, and hence may be more motivated to view/read yaoi materials due to the female-oriented nature of this genre [1]. By contrast, male yaoi enthusiasts yielded higher scores on the Identification/ self-analysis and Pure escapism/lack of reality motive factors. Male participants’ higher score on the Identification/self-analysis dimension supports previous qualitative studies analyzing heterosexual and gay male readers [13, 16–18], who may seek in yaoi representations of men like themselves—no matter how unreal the narratives about their romantic lives may be—or a temporary escape from demanding masculine gender-role expectations.

In addition, Dislike for standard romances/shoujo, Pure escapism/lack of reality, and Pure “love” without gender & pro-gay attitude factors appeared to be important motive dimensions. It is also notable that non-heterosexual respondents to this survey scored higher than heterosexual respondents on all motive dimensions except for A female-oriented romantic/erotic genre. A cross-cultural study involving 25 European countries revealed that the highest perceived lesbian, gay and bisexual (LGB) acceptance was observed in Denmark, Netherlands and Sweden, whereas the lowest level of acceptance was reported in Ukraine and the Russian Federation. Hungary was the 19^th^ most accepting country of the 25 European countries [40], reflecting a relatively low level of acceptance. This culture-specific characteristic may explain why heterosexual Hungarian yaoi enthusiasts seem to prefer yaoi’s sexual and aesthetic aspects over its perceived or actual pro-gay messages, but non-heterosexual readers scored comparatively higher on it. It is possible that non-heterosexual viewers identify with yaoi characters more easily than heterosexuals and consume yaoi to resist or temporarily escape a heterosexist culture.

The Pure entertainment dimension suggests that yaoi serves a function consistent with that of other media; that is, the entertaining and relaxing aspects of media consumption also appeared in studies exploring the motives for using mass media (e.g., television viewing) and interactive media (e.g., online gaming) [41, 42].

The Melodramatic/emotional elements dimension of yaoi was found to be relatively unimportant. This is somewhat surprising, given previous research in the U.S. suggesting that the slow but consistent development of a romantic relationship is an important part of yaoi’s draw for many readers [19]. Further research seems necessary to determine whether this may be a culturally specific difference. The Melodramatic/emotional elements dimension was also weakly negatively associated with age, indicating that older yaoi consumers are less likely to view/read yaoi for its portrayal of intense emotions. One possible explanation for this finding could be that younger yaoi consumers have higher levels of sensation-seeking compared to older consumers, as was proposed by Vettehen and Nuijten [43] in relation to motivations for viewing television news stories.

The results of the present study demonstrated measurement invariance across gender and sexual orientation, indicating that group comparisons between male and female or heterosexual and non-heterosexual yaoi viewers/readers with regard to their yaoi media consumption motives are meaningful, since no substantial differences were observed either in the factor structure, factor loadings or the intercepts between male and female or heterosexual and homosexual participants.

In summary, the present study provides a more nuanced picture of the motives underlying yaoi media consumption and offers a comprehensive, multidimensional instrument to assess them in a reliable way. However, this study has some limitations that should be considered for future research. First, the present sample is not representative of the population of yaoi media consumers, so the results of this study may not be generalizable to all yaoi viewers/readers. Furthermore, this model has been developed primarily within a Hungarian and U.S. context and therefore reflects values and behaviors that may be particular to those cultures. Further expansion of this model by researchers in other countries who can take into account motivations that might not have arisen in this model’s initial development is highly desirable.

Second, the convergent and divergent validity and test-retest reliability of the developed assessment instrument should also be examined in future research. Finally, due to the large variety of yaoi media forms (e.g., anime, manga, and video games), certain items of the YCMQ may not be applicable to all forms of yaoi media consumption (e.g., some items of the Art and aesthetics to drama CDs). However, the primary aim of the development of the YCMQ was to create an assessment instrument that assesses motives for consuming yaoi content in a wide range of related media.

Despite these limitations, the findings of present study support previous qualitative research on yaoi consumption and provide researchers with a basis for further quantitative research on yaoi media consumption motives. The Yaoi Consumption Motives Questionnaire (YCMQ) demonstrates strong psychometric properties and proves to be an adequate assessment instrument to assess the considerably full range of yaoi media consumption motives.
